# Exploratory Study on Individual Locomotor Activity in Local Dual-Purpose and Commercial Breeder Pullets

**DOI:** 10.3390/ani13182879

**Published:** 2023-09-10

**Authors:** Pia Schürmann, Senta Becker, E. Tobias Krause, Sonja Hillemacher, Wolfgang Büscher, Inga Tiemann

**Affiliations:** 1 Institute of Agricultural Engineering, Agricultural Faculty, University of Bonn, 53115 Bonn, Germany; pia.schuermann@uni-bonn.de (P.S.); sonja.hillemacher@uni-bonn.de (S.H.); buescher@uni-bonn.de (W.B.); inga.tiemann@uni-bonn.de (I.T.); 2 Institute of Animal Welfare and Animal Husbandry, Friedrich-Loeffler-Institute, 29223 Celle, Germany; tobias.krause@fli.de

**Keywords:** animal welfare, general locomotor activity, breeder, dual-purpose chicken, local breeds, gallus gallus, pullets, personality

## Abstract

**Simple Summary:**

Animal welfare is becoming increasingly important in the transformation process of poultry production. Welfare is closely linked to animals’ ability to be active and to express their natural behavior; since activity promotes physical health, it is associated with positive welfare. Currently, dual-purpose chickens are presented as a problem-solving strategy for the culling or rearing of males from layer lines. However, for these breeds, data welfare-associated behaviors, such as activity, are lacking. Activity is predominantly associated with locomotion and foraging, reflecting major traits of natural behavior. Here, we show that locomotion varies greatly between breeds and individuals. Our results also support the idea that animals show individual behavioral patterns, which can also be quantified as unique personality traits. Individuals showing maximum activity cover twice the breeds’ mean range. We suggest the implementation of more precision-livestock systems in poultry research and rearing to achieve more individual data, offering opportunities to understand the mechanisms of animal–environment interactions. Finally, these findings can be used not only for breeding, but also for adapting housing environments and management to increase (individual) animal welfare.

**Abstract:**

Improving animal welfare is a prerequisite for the societal acceptance of poultry production. Support for improvements requires practical tools to quantify animal welfare and identify predispositions at the individual level, where possible. In this study, the activities and behavior of dual-purpose chickens (N = 245) and commercial breeders (N = 224) were analyzed. The general locomotor activity (GLA) data were collected using an RFID system over five days with 9-to-14-week-old animals. The results show that the animals of comparable age and stocking density differed from each other in their activity (*p* ≤ 0.001) according to breed, but no sex differences were observed (*p* = 0.159). No correlations were found between GLA and plumage condition (*p* > 0.05). The individual variations within the breeds are presented and discussed on an animal-by-animal level, providing new insights into the individual behavioral variability of chickens. The RFID systems can reliably generate GLA data that help to understand the potential interplay between behavior and animal welfare. The technology is also suitable for creating individual (personality) profiles that can be used for breeding. With a better understanding of the role of activity, husbandry and management practices can be adapted to improve animal welfare.

## 1. Introduction

The ban on the killing of day-old male layer chicks, e.g., in France and Germany in 2022, has led to the search for alternatives to rearing these males from laying strains, especially regarding the genotypes used for production [1,2]. Dual-purpose chickens are among these alternatives, with cockerels used for meat production and hens used for egg production. This approach follows the interest of German customers, of whom 50% support the idea of raising cockerels and pullets together [3]. The participants in this study mostly had a higher level of education and belonged to the age group of 46 to 65. In turn, local dual-purpose breeds have also gained attention in research [4] and seem to be a compromise in relation to high-performing birds, at least for niche markets and regional production [5]. Combined with the development of commercial dual-purpose hybrid lines, a variety of genotypes is available for production on a commercial scale. However, some of these breeds might not be adapted to living in large groups and intensive husbandry systems [6], which may affect their welfare. Generally, animal welfare is not only defined as the absence of health impairments, but should also involve the presence of a positive mental state [7,8]. Currently, the measurement of (positive) animal welfare is a challenge, as direct measurements of animal welfare are difficult to obtain, which is why, usually, indicators of animal welfare are required [9]. One such indicator is behavior related to either welfare problems or positive welfare [10]. Behavioral indicators can be used to indicate welfare problems, such as reactions to, e.g., toe or feather pecking [11,12,13,14], or as positive indicators of, e.g., reduced anxiety or better cognitive skills in improved housing [15,16,17,18]. In addition, behavior is a crucial readout parameter reflecting the adaptive capacity of an animal [19].

One behavioral indicator for animal welfare is the general locomotor activity (GLA), which varies between individual chickens and among breeds and is this influenced by genetics [20]. Activity can be defined as locomotion behavior covering walking, running, jumping, flying activities, and, in general, foraging and exploration [21]. The GLA can be described as an individual’s locomotor activity in their home-pen area while performing undisturbed behavior, and it was shown to be a heritable trait [22]. One behavioral factor linked to GLA is fear. Selected hens with lower GLA showed less fear than those with higher GLA [22]. Furthermore, production traits may interfere, as chickens from a high-GLA line were significantly heavier at 30 days of life than those in a low-GLA line, although this effect disappeared in adulthood [22]. The opposite was found in quail, with fear expressed less in birds with high GLA [23].

In addition, GLA is linked to other welfare-related behaviors, such as feather pecking [11] and exploration [24,25]. Furthermore, in rearing broilers, activity is an important factor, as it reflects a good welfare status. Activity has been found to be more pronounced in slow- than in fast-growing broilers [26]. The selection of broilers for activity early might have a positive effect on mobility later in life and, therefore, on leg health [27]. The assessment of activity at flock level has been performed based on video data using, e.g., optical flow [28] or the increasingly widespread techniques of deep vision [29]. Due to the small body sizes of the animals and their natural use of three-dimensional structures, tracking individual chickens in farm settings is challenging. Only a few studies in this area have been successful, such as those using pedometers [30] and RFID techniques [31]. For measuring GLA, Kjaer [20] designed an experimental setup using passive RFID tags on their animals. The tags started recording once the animals crossed an antenna. These antennae were distributed within a pen and covered by litter. Transponders use unique identification codes, which allow antennas to register the locations of multiple transponders simultaneously [32]. These RFID systems can only register the presence of animals, and not their specific behavior. They are used, for example, to track broilers from hatching onwards for range use when installed in front of pop holes [32,33]. The assessment of an individual’s behavior within its group does not only provide more insights into individual chickens’ behavior, but also into an animal’s personality. The consistency of behavioral patterns over time is the basis for animal personality [22,34]. In addition, GLA patterns might contribute to the personalities of individual chickens.

In this study, two German local dual-purpose breeds, Bielefelder (BIE) and Ramelsloher (RAM), and two breeders of commercial hybrids, Ranger (RA; the parent stock for the slow-growing broiler, Aviagen) and White Rock (WR; the parent stock for the brown layers, Lohmann Breeders), were investigated regarding their GLA. Additionally, the wild ancestor of the domestic chicken, the Red Junglefowl, was evaluated [35]. The offspring of the RA and WR are used as hybrids in commercial poultry farming and, therefore, underwent a far more intensive domestication process compared to the local dual-purpose lines. The breeders are of interest because maternal effects, e.g., via hormones, are an important factor in early-life behavioral development in commercial hybrids, and physiological maternal stress creates the risk of developing anxiety and maladaptive behavior, like feather pecking [36].

The aim of this study was to investigate the variation between individuals within a breed regarding their GLA. Although welfare indicators are typically not specifically designated for pullets, we used these as proxies to relate the individual GLA data to individual animals’ welfare indicators. For the dual-purpose chickens, breed and sex differences were also investigated, as they were reared in mixed-sex environments, the whereas breeder hens were reared in a single-sex context. The age and small group size of the Red Junglefowl prevented them from being compared to other breeds and lines. Instead, they served as a reference.

## 2. Materials and Methods

### 2.1. Animals and Housing Conditions

**Figure 1:**
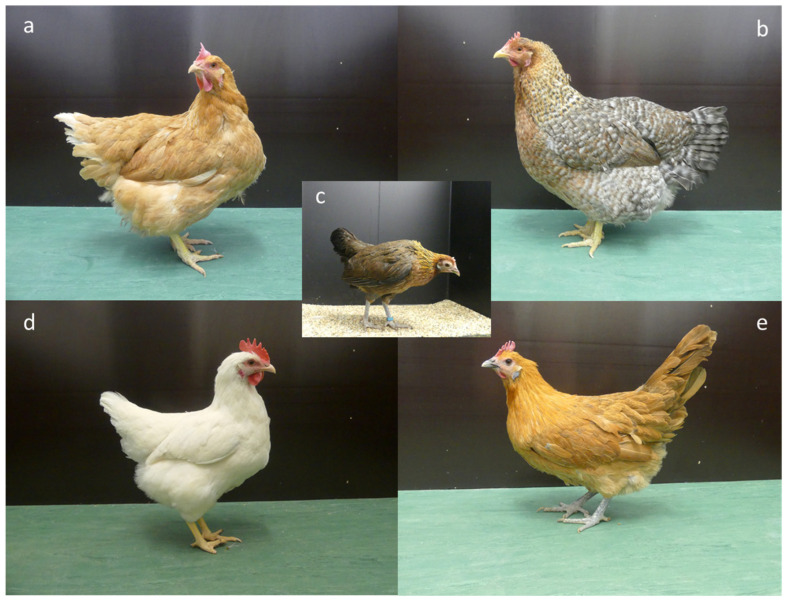
Female phenotype of the investigated breeds: Ranger (**a**), Bielefelder (**b**), Red Junglefowl (**c**), White Rock (**d**), and Ramelsloher (**e**).

For this study, local dual-purpose breeds (BIE and RAM) and breeder stocks for slow-growing broilers (RA) and layers (WR) were investigated. The BIE and RAM were hatched on a farm (Campus Frankenforst at the Faculty of Agriculture, University of Bonn (Königswinter, Germany)). This resulted in different numbers of animals due to different hatching rates. In addition, these breeds were reared in mixed-sex groups consisting of females and males (see Table 1 for sex ratios). The breeder stocks were obtained from a commercial hatchery with the support of the respective breeding companies, and they consisted of females only, as these were already sexed at the commercial hatchery, and only females were reared. Animals were housed separately by breed/line. One group of each breed/line was observed. Figure 1 shows the phenotypes of the breeds.

The home pens were identical, measuring 15.4 m^2^ (3.97 × 3.88 m), and with one window offering daylight (11.9% in relation to the floor). Only the RA were housed in a pen measuring 24.88 m^2^ (6.13 × 4.06 m), with two windows (9.6% daylight in relation to the floor). All pens were equipped with perches (1.80 × 0.5 m). The pens corresponded to a conventional floor system, with water nipples and round feeders. All chickens were fed a biological pullet feed during the rearing period (pullet developer, “Maxima 9–17”; crude protein 17%; methionine 0.3%; calcium 0.8% phosphorus 0.59%; 11.8 MJ ME/kg; without coccidiostats; Reudink B.V. (NL-7241 CW Lochem)). Except for the RA and WR groups, all groups had ad libitum access to feed and water. The RA and WR were fed according to the management guide for breeders. The pens were equipped with manual ventilation systems and the floor was covered with wood shavings (Allspan^®^ Classic, Allspan Spanverarbeitung GmbH, Karlsruhe, Germany) as bedding material. Furthermore, pecking stones and alfalfa bales were given to support natural behaviors. According to the veterinarian in charge of the flock, vaccinations of the chickens followed a standard procedure for the rearing period. Table 1 summarizes the ages, group sizes, stocking densities, average weights, and sex ratios for the sampled animals.

### 2.2. Red Junglefowl

In addition, Red Junglefowl were studied. These were only available as adults in a breeding group on campus at the time of data collection, so they were considered separately from the other breeds. The Red Junglefowl was the only group with access to an outdoor area, which measured 29.2 m^2^ (7.30 × 4 m), attached to an indoor floor housing measuring 15.33 m^2^ (7.30 × 4 m). The pen was equipped with perches and adult Red Junglefowl were provided with nests (0.5 × 0.45 m). They received an ecological layer diet (laying mash; crude protein 15.6%; methionine 0.29%; calcium 3.6% phosphorus 0.59%; 11.3 MJ ME/kg; without coccidiostats; Reudink B.V. (NL-7241 CW Lochem)).

### 2.3. Data Collection

**Figure 2:**
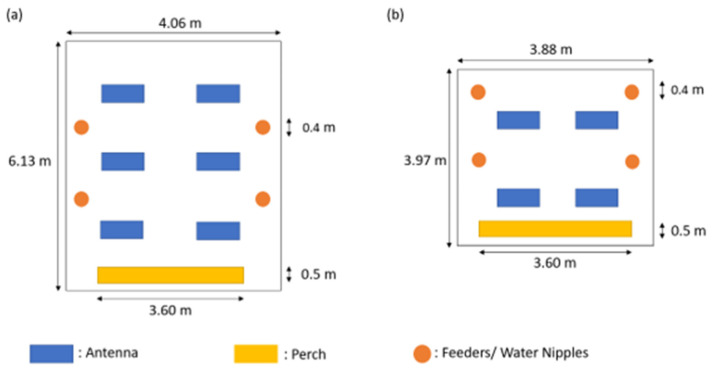
Experimental setup of antennas covering the pen. Positions of antennas, perches, feeders, and water nipples and dimensions of pens are given. The RA were housed in compartments shown in (**a**) and the breeds BIE, RAM, and WR were housed in compartments as shown in (**b**).

We measured the chickens’ general locomotor activity (GLA) using Kjaer’s 2017 study design [20]. The data collection was conducted in the home pens for five days, continuously (light period 14.5 h per day). Measurements were carried out from Thursday to Monday. Only feeding and animal-care routines, which lasted around 15 min a day per pen, were carried out to minimize human contact. Every chicken was marked at hatching with an individually numbered wing band. Before recording, the sexes and weights (body weight = BW, ±1 g) of the chickens were determined. Additionally, each chicken received a leg band with a transponder (approx. 0.5 g, 2 mm diameter, and 10 mm length). Every transponder had a unique number, which was recorded when the bird was approximately 15 cm from the antenna. Four antennas (each measuring 76 × 30 × 10 cm) were placed in a circular pattern on the floor, with equal distances between them and the wall. The antennas covered 18.7% of the pen‘s floor, including the radius within which the antennas still registered the transponder. Areas out of the antennas’ reach, e.g., perches and nests, were excluded. The RA and the Red Junglefowl had six antennas due to their different pen sizes, which covered similar floor areas of 16.5% for RA (see Figure 2) and 27.3% for the Red Junglefowl. The antennas were then covered with litter. Twice per second, the system (Ganter Pigeon Systems GmbH, Schruns, Austria) scanned the antennas and recorded the transponder number (bird’s identity) within a 15 cm radius around the antennas, along with antenna location and time and date of the recording day. Thus, the system recorded the frequency of antenna crossings per animal, which was counted as GLA [20]. At the end of the recording, the results were given as the total amount of registered contact events between every individual chicken and the antennas per day. This technique was established by Kjaer [20]. Due to the limited number of available antennas, breeds were observed at different ages.

Parts of the Welfare Quality^®^ Assessment protocol (WQA) for laying hens were applied [37] to assess the welfare of the pullets, including plumage conditions, footpad lesions, keel-bone damage, and comb injuries. Plumage condition was recorded separately for the head, back, and cloaca regions. The week before and after the experiment, 15 chickens from each line were selected randomly and evaluated according to the WQA. Sampling for RA breeders was conducted the day before recording the GLA. For WR, the data were collected one week after the recording of activity. Due to the stressful handling of the (wild) Red Junglefowl, no welfare data were collected.

### 2.4. Statistical Analysis

For the statistical analysis, the program SPSS^®^ Statistics 28 was used (IBM Corporation, Armonk, NY, USA). The median (25th, 75th percentiles), range (*R*), and minimum (Min) and maximum (Max) were calculated. Significance level α was set, with *p* ≤ 0.05 being significant, *p* ≤ 0.01 very significant, and *p* ≤ 0.001 highly significant. All data were tested for normal distribution. Since they were not normally distributed, to calculate correlations between activity and welfare parameters, the Spearman–Rho correlation was used. The correlation coefficient R was set with |R|= 0.10 as low correlation, |R|= 0.30 as moderate correlation, and |R|= 0.50 as high correlation.

To exclude the effect of age, only WR and RA were compared to each other, as well as BIE and RAM, as they were observed at the same ages (weeks of life). Furthermore, since there was a contrast in hatching conditions (commercial hatchery vs. on farm), the groups were not compared with each other, but only within the subcategories (breeders: WR to RA and dual-purpose: BIE to RAM).

The Mann–Whitney-U test for independent samples was used to compare the GLA of WR to RA, of BIE to RAM, and sexes within the local dual-purpose breeds. Levene’s test was used to investigate the equality of variances in GLA between the breeds. Activities of all breeds were recorded and analyzed at the individual-animal level.

## 3. Results

### 3.1. Individual Differences in GLA

**Figure 3:**
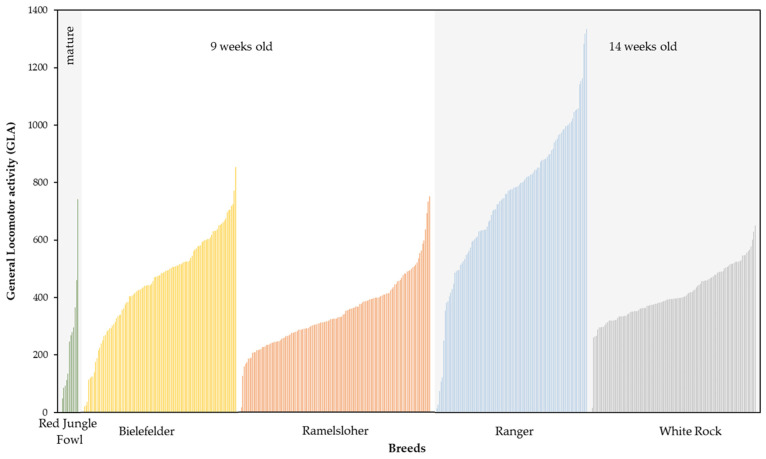
General locomotor activity (GLA) for the individuals of the investigated Red Junglefowl, Bielefelder (BIE), and Ramelsloher (RAM), and as well as the breeders Ranger (RA) and White Rock (WR). Each bar represents one individual from the breed. The different age groups and comparable stocking densities, i.e., groups that were statistically compared, are indicated by different background colors. The GLA expresses the number of antennas passed per bird and day during five days of recording.

Large individual differences between the animals within each breed were registered. The individual GLA of each animal can be seen in Figure 3, by breed. Above all, Figure 3 shows the large dispersion of the individual animals’ GLA. Although breed-related patterns are visible (e.g., RA), there were large dispersions of the individual animals within the breeds. Overall, the RA showed the highest individual activity, with a median of 777.0 (598.0, 899.0), and the largest dispersion, with a range of *R* = 1321, with Min = 14 and Max = 1335. The BIE had the second-highest activity, with a median of 477.0 (348.0, 578.5) and dispersion with a range of *R* = 834, with Min = 20 and Max = 854. The lowest activity was shown by the RAM, with a median of 326.0 (265.5, 408.0) and a dispersion with a range of *R* = 734, with Min = 17 and Max = 751. The lowest dispersion was shown by the WR, with a median of 395.5 (349.75, 478.25) and a dispersion with a range of *R* = 641, with Min = 10 and Max = 651. The Levene test based on the median showed that the variances in the GLA were not equal among the breeds (F(3466) = 26.362, *p* ≤ 0.001).

### 3.2. GLA and Animal Welfare

For all the groups, the animal-welfare data were collected through a sub-sample, in addition to the activity measurements. The plumage conditions, footpad lesions, keel-bone damage, and comb injuries were adapted from the WQA, although these are not specifically designated for pullets. Due to singularities, no correlations with the activity data were calculated for the parameters of footpad lesions, keel-bone damage, or comb injuries because there were no scores > 0. For plumage condition, however, damages were found in the RAM only in the back and cloacal area, with no significant correlations shown between the GLA and plumage condition for either the back area (N = 21; r = −0.091; *p* = 0.696) or the cloacal area (N = 21; r = −0.080; *p* = 0.729). Furthermore, for all the other breeds, no significant correlations between plumage condition and activity were found (all *p* ≥ 0.05).

### 3.3. Breed, Sex, and Weight Differences

**Figure 4:**
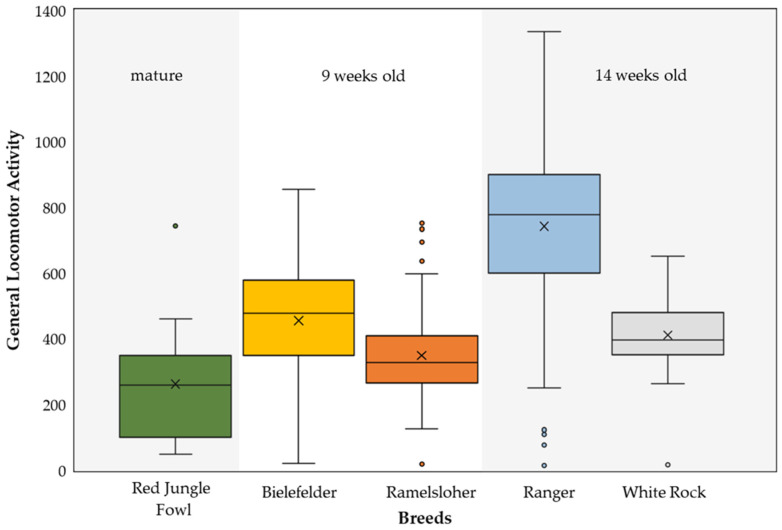
Comparison of general locomotor activity (GLA) distribution for the investigated breeds, Red Junglefowl, Bielefelder (BIE), and Ramelsloher (RAM), as well as the breeders Ranger (RA) and White Rock (WR). The different age groups and comparable stocking densities, i.e., the groups that were statistically compared, are indicated by different background colors. The upper and lower boundaries of the box indicate the upper (75%) and lower (25%) quartiles, the median is shown by the line within the box, the mean is indicated by the cross inside the box, and the whiskers show minimum and maximum values. Outliers less than or equal to 1.5 times the interquartile range are presented with dots.

The breed differences were investigated further, since breed patterns and individual differences were found (Figure 4). To exclude the effects of age, only the WR and RA were compared to each other, as well as BIE and RAM, as they were observed at the same ages (weeks of life). The breeders showed significant differences in GLA (U = 1293.0; *p* ≤ 0.001), with the RA showing a higher GLA compared to the WR. In the local dual-purpose breeds, the BIE showed higher GLA compared to the RAM (U = 4127.0; *p* ≤ 0.001). Furthermore, in the local breeds, sex differences were evaluated, since the animals were reared in a mixed-sex environment. The cockerels and pullets from the local dual-purpose breeds did not differ in their GLA (U = 8104.0; *p* = 0.159). Within the breeds, correlations between the GLA and the weights of the animals were also calculated, but no significance was found (all *p* ≥ 0.05). The Red Junglefowl were excluded from the statistical analysis because of the major differences in age and housing system.

## 4. Discussion

The animals investigated in this study showed significant individual variations within each breed, i.e., within dual-purpose breeds as well as within breeders. Some of the animals showed high levels of activity and locomotion in their home pens, while others showed less activity. The variation in RA was higher compared to the WR, BIE, and RAM, since the local breeds showed lower activity levels than the commercial breeders. In the RA, the activity increased 95-fold from the individual showing the lowest activity to the individual showing the maximal activity. In the WR, the maximal activity was 65 times that of the minimal activity. In the BIE and RAM activity range was a maximum activity level that was 43 and 44 times that of the minimum level, respectively.

Activity measurements have gained increasing interest in poultry research. However, activity measurements are more typically carried out in open fields or in connection with outdoor use than in indoor floor-rearing systems. For example, some animals spend long periods foraging and make greater use outdoor areas, while other animals show this behavior only to a smaller extent [34]. Kjaer [11] also describes how higher activity levels lead to more pronounced exploration and, thus, foraging behavior. In the local dual-purpose lines, the BIE showed a higher GLA than the RAM. The average weight of the BIE was also higher than that of the RAM, which is consistent with the findings of other studies: more active chickens show higher weights [11,22]. The cockerels and pullets showed no differences in their GLA. By contrast, when examining flocks at the fifth week of age, Kjaer [20] showed that in four out of six generations, males were more active in general. Our results did not support these findings; therefore, there might be line- or even generation-specific differences. In open fields, chickens showing high levels of activity, meaning prolonged track lengths, might show increased exploration behavior [38]; alternatively, prolonged track lengths might account for increased fear [39]. This topic is still under discussion [24]. Tiemann et al. [24] showed that locomotor activity in open fields is breed-specific and, therefore, influenced by genetics. Even within breeds, genetic polymorphism can lead to individual differences [40]. Beyond the breed level, recent studies demonstrate that animals show individual behavioral patterns [34,41].

Generally, individual differences expressed in consistent patterns over time and context add to the personality profiles of species [42]. The study of the personalities of chickens can improve animal welfare by allowing breeding objectives, management, and environments to be adjusted accordingly [43]. To determine whether a personality profile can be mapped based on an activity, consistent behavioral patterns should be established [34]. Here, we approach this topic with a snapshot using individual data from just one week of the animals’ lives. Prolonged observation within the pen, in addition to possible further individual experimental tests, could contribute to answering the question recently posed by Ferreira et al. [34] regarding personality traits in chickens. The collection of GLA data and their integration into the selection of breeding animals based on personality differences could help to enhance the adaptability of animals. Furthermore, Krause et al. [22] observed that chickens with higher GLA rates were more fearful than the lines with lower GLA rates. This observation suggests that GLA is also correlated with other behavioral patterns. Since activity and fear are important factors in assessing animal welfare, collecting individual data can not only help in creating personality profiles but also in selecting chickens with higher or adapted activity levels.

The selection of higher activity levels could lead, for example, to the breeding of more mobile chickens, with a greater allowance of, for example, foraging and exploration time. Such activity would help to reduce several animal-welfare concerns, such as leg disorders or lameness [27]. When comparing the breeders, the RA showed significantly higher activity than the WR. One reason for this finding could be the difference in stocking density or the (restrictive) feeding management of the RA to obviate skeletal disorders resulting from uncontrolled weight gain and the associated increase in foraging behavior [44]. This is especially important since GLA is a heritable trait, with h^2^ from 0.33–0.38 [20]. The linkage between individual GLA and welfare traits will be an interesting research question for the future.

In addition to individual differences in activity, the animals were also examined in terms of other welfare parameters. In general, it can be stated that the animals’ welfare status was high. The breeders showed no signs of feather pecking, even though they were hatched commercially, which is associated with an increased risk of feather pecking [45]. The influence of individual behavioral patterns cannot be neglected when considering animal welfare at the flock level, especially if animal-welfare parameters are to be correlated with behavioral activity patterns [41,46,47]. White Leghorn lines selected for their higher levels of locomotor activity showed more feather pecking [11]. Furthermore, other studies showed that young White Leghorns who performed more foraging and exhibited fewer resting and dustbathing behaviors were more likely to perform severe feather pecking as adults [48]. Nevertheless, chickens selected for their higher and lower GLA based on a New Hampshire genotype showed no differences in feather pecking [22]. Among the local lines, however, the RAM showed damages in plumage, although no significant correlation between the plumage condition of an animal and its activity could be found. The small sample size and the ages of the pullets may have been a limiting factor here, i.e., individual (WQA) data should be of greater importance in the future.

However, the small social group and environmental enrichment (pecking stones and alfalfa bales) could have led to the expression of more diverse behaviors, potentially improving welfare [49]. However, Baxter et al. [50] did not find an effect of enrichment on general activity. In addition, the Red Junglefowls’ small sample size, due to the limited availability of individuals, should be considered. Nevertheless, the usually more exploratory Red Junglefowl were provided access to an outdoor area, where no GLA measurements were taken, which might have resulted in higher actual GLA figures than those recorded [46]. Another reason could have been the intensive breeding of the breeder lines compared to the local dual-purpose breeds, resulting in consistent scores in the welfare assessment. Therefore, production traits might layer GLA with other welfare-associated behaviors [51,52,53].

Local dual-purpose breeds are typically not used for conventional food production, but are rather kept in small groups consisting of one rooster and up to five hens with access to a free range [54]. Changes to commercial production systems might produce new social environments for these chickens [54]. Such changes might also be reflected by differences concerning animal welfare, especially in plumage damage, as in this study. Breeders are used for breeding and are kept in commercial reproduction farms [36]. Although this study did not find a connection between animal-welfare measures and activity, other studies indicate the opposite [47]. Activity may serve as a potential animal-welfare indicator. For example, broilers with higher levels of locomotor activity show improved skeletal conditions [55]. Bessei [56] stated that locomotor activity is important for bone ossification. As this activity can also affect bone stability [57], a continuous ability to express movement is desirable as a high animal-welfare standard. In this way, activity would not only play a role in the rearing of broilers, but also in the rearing of pullets and dual-purpose chickens. The level of activity contributing to a higher welfare state might therefore be different between broiler and layer lines and should be considered not only species-specific, but also line-specific.

This study did not cover all the potential aspects that could contribute to differences in GLA expression. One reason for the large variability in the GLA between the individuals might have been dominance, with the more dominant individuals gaining more access to food, making dominance advantageous in fitness-related outcomes [58]. Thus, GLA may indicate other behaviors, which probably include some that relate to the social status of the individual animals. Furthermore, a diurnal rhythm may underly the GLA, which would be interesting to address in future analyses. Future studies should expand the sample sizes used for the animal-welfare data and investigate the gait score to allow more accurate conclusions about the relationship between animal welfare and activity.

The variability within the breeds indicates the large range of individuality among the animals. In general, it must be considered that the RFID system only registers GLA movements from one antenna to another and no other behaviors [32]. Nevertheless, GLA can be used to effectively track the activity of a group. The main advantage of this is that the activities of animals are recorded in the animals’ home pens without any human influence, which reduces the risk of biased results [22]. In addition, the recording of an individual’s behavior provides valuable insights into their behavioral patterns, adding value to the individual observations of the animals. The implementation of more RFID readers into areas like nests and dust baths, as well as combining the system with automatic feeding and drinking scales, could generate more data to predict health conditions and shift the consideration of welfare from the group to the individual level [59,60].

## 5. Conclusions

The results of this study show that within-breed individuals show wide variability regarding their GLA. No correlations between GLA and plumage condition were found in this study, but further research should be conducted in this area. While the breeds differed from each other regarding their GLA, the sexes did not. In combination with more knowledge about GLA, the implementation of RFID systems in pens to track activity might be beneficial in the future in order to understand the complexity of animal welfare and behavior, and it would be valuable for selection and breeding. In particular, animal-specific differences provide insights into personality profiles and show that the technology-assisted tracking of behaviors offers not only insights into welfare-related behavioral profiles, but also a starting point from which to improve husbandry and management, aiming to optimize individual animal welfare, including among chickens.

## Figures and Tables

**Table 1:** Overview of the examined lines and animals, including age (weeks of life), group size, number of sampled animals, stocking density (animals m^−2^), average weight (g), and sex ratio at the time the measurements took place. Group and sample sizes differed due to experimental setup and technical difficulties during data collection, with animals losing/ removing their RFID-Tags.

Line	Age (Weeks of Life)	Number of Birds (n)	Sampled Animals (n)	Stocking Density (Animals m^−^²)	Average Weight (g)	Sex Ratio (m:f)
Bielefelder (BIE)	9	112	109	7.27	967	1:2.6
Ramelsloher (RAM)	9	152	136	9.87	742	1:2.2
Ranger (RA)	14	125	107	5.02	1371	f only
White Rock (WR)	14	125	117	8.12	1117	f only
Red Junglefowl *	116	12	12	0.78	601	1:4

* Due to low sample sizes and ages, Red Junglefowl were not compared statistically to other breeds, but were included descriptively as ancestral reference.

## Data Availability

The data presented in this study are available on request from the corresponding author.
